# Development and assessment of the psychometric properties of a compassionate care questionnaire for nurses

**DOI:** 10.1186/s12912-021-00691-3

**Published:** 2021-10-07

**Authors:** Banafsheh Tehranineshat, Mahnaz Rakhshan, Camellia Torabizadeh, Mohammad Fararouei, Mark Gillespie

**Affiliations:** 1 Community Based Psychiatric Care Research Center, Department of Nursing, School of Nursing and Midwifery, Shiraz University of Medical Sciences, Shiraz, Iran; 2 Department of Epidemiology, Shiraz University of Medical Sciences, Shiraz, Iran; 3 School of Health Nursing and Midwifery, University of the West of Scotland, Paisley, Scotland UK

**Keywords:** Compassion, Nursing care, Questionnaire development, Psychometric properties, Nurses

## Abstract

**Background:**

Compassionate care is emphasized within professional ethics codes for nursing and is a key indicator of care quality. The purpose of the present study is to develop and assess the psychometric properties of a compassionate care instrument for nurses.

**Methods:**

This methodological study was carried out in two phases -qualitative and quantitative-from February 2016 to October 2018. In the qualitative stage of the study, a content analysis approach was used to establish the concept of compassionate care through interviews with nurses, patients, and family caregivers. The initial draft of the questionnaire was developed based on the qualitative findings and a subsequent review of the literature. In the second phase, the psychometric properties of the questionnaire were assessed for validity and reliability. Data analysis was performed using descriptive and inferential statistics in SPSS v.16.

**Results:**

From the results of the qualitative phase and review of literature, 80 items were extracted. In the quantitative phase, after evaluation of the face and content validity, 40 items were kept. After measurement of the construct validity, 28 items whose factor loading was above 0.4 were retained. Measurement of convergent validity showed a moderate correlation between the questionnaire and the nurses’ caring behaviors scale (r = 0.67, *P* = 0.01). The reliability of the 28-item questionnaire was tested by measuring its Cronbach’s alpha coefficient and intra-class correlation coefficient which were found to be 0.91 and 0.94 for the whole questionnaire, respectively.

**Conclusion:**

The questionnaire has enough validity and reliability to be used for measuring the nurses’ compassionate care. Therefore, the instrument can be used to measure and record the quality of nursing care.

**Supplementary Information:**

The online version contains supplementary material available at 10.1186/s12912-021-00691-3.

## Background

Compassion is the main focus of health care policies and an essential characteristic of person-centered nursing care [1–3]. It is widely considered as the first principle of health care ethics [4] as well as the basis of high-quality care delivered by healthcare professionals [5].

Frampton et al. (2013) define compassion as “a deep sense of connection to the experience of human suffering that requires personal awareness of others’ suffering and moral response” [6]. From Dewar’s perspective, compassionate care is the relief of individuals’ suffering [7]. Several studies have reported positive clinical and health outcomes for compassionate care in both patients and nurses. For example, compassionate care can increase the patients’ satisfaction and nurses’ job satisfaction. On the other hand, lack of compassionate care leads to lower standards in care [8, 9].

Compassionate care is considered as the patient’s right [10] and is one key aspect of the professional performance standards that health care providers need to be educated about and healthcare systems should measure and report [11]. Although ethical principles, including compassion, are always emphasized in the educational context, the real problems arise when nurses face organizational realities [12]. Providing compassionate care depends on not only the therapist, but all the members of the healthcare team and the organizational context [13].

One of the major barriers to improving the quality of patient care and satisfaction with care is lack of a compassionate clinical care scale with strong psychometric properties [14]. Currently, there is not a standard instrument for measuring compassionate care in the health care system. Compassion is one of the significant aspects of the quality of care and should be continuously evaluated [15].

Assessment of compassionate care is essential for evaluating and enhancing clinical performance [13]. Empirical evidence of attention to compassionate care in health care systems is scarce since a practical perception of the nature of compassion has not been well developed. In addition, most previous studies are based on predefined theoretical definitions that lack specificity, clinical applicability, and conceptual validity and are not patient-orientated [11]. Also, few studies have been conducted using appropriate compassionate care tools in nursing.

One of the challenges in measurement of compassionate care in nursing is that the meaning of compassionate care varies depending on people’s perspectives. The concept of compassion is complex and its measurement needs to reflect the concept from the perspective of patients, their family members, and the clinical staff [16].

There are some instruments which measure compassionate care in physicians [17] and the public, compassionate competence [18, 19] and non-verbal compassionate communication [20]. However, in most available tools, the definition of compassion has been borrowed from a dictionary or a review of literature. Therefore, they do not cover all aspects of compassionate care as delivered by nurses. The present study aims to design and evaluate the psychometric properties of an instrument for measuring the nurses’ compassionate care.

## Methods

### Study design

The present study used an exploratory sequential design to develop an instrument in two phases- qualitative and quantitative-from February 2016 to October 2018 [21]. The study was conducted in one of the major cities in the south east of Iran. The objective of the qualitative phase was to identify the concept of compassionate nursing care and its dimensions and sub-dimensions based on the experiences of nurses, hospitalized patients and their family caregivers. In this phase we used the conventional content analysis approach recommended by Graneheim and Lundman (2004). Content analysis approach was used to interpret the content of textual data to gain a deep understanding of the concept of compassionate nursing care [22]. After the meaning of compassionate nursing care and its constituent parts was established, the researchers used the blueprint to create the initial pool of questions based on the categories and subcategories extracted from the definition of compassionate nursing care. Subsequently, an extensive review of literature was conducted to complete the items of the questionnaire. Thus, the initial draft of the questionnaire was created. In the quantitative phase, the psychometric properties of the questionnaire, including its validity and reliability, were assessed. The validity of the questionnaire was measured according to its face validity, content validity, and construct validity. In the quantitative phase, COSMIN (Consensus-based Standards for the selection of health Measurement Instrument) criteria, consisting of 9 measurement properties in the three domains of reliability, validity, and responsiveness, were employed to evaluate the psychometric properties of the compassionate nursing care questionnaire [23].

### Phase 1. Qualitative study

In the qualitative phase of the study, 20 nurses (18 clinical nurses and 2 nurse instructors), 8 patients, and 6 family caregivers were selected via purposeful sampling and according to the inclusion criteria from various departments (internal, surgical, emergency, CCU, ICU, and hemodialysis) of university hospitals. After being selected, the subjects were interviewed individually. In addition, two focus group interviews were conducted with one group consisting of 6 nurses from internal, emergency, CCU, and ICU departments and another consisting of 6 nurse instructors.

The inclusion criteria for the nurses were having at least a bachelor’s degree in nursing; working in a fixed ward; not being in charge of a critically-ill patient; suffering from physical or emotional fatigue as a result; having manageable workload and appropriate physical and mental status, and being prepared for a 45-min interview as confirmed by the interviewee.

The inclusion criteria for the patients were being over 18 years old, being hospitalized at least for 3 days, being in good physical conditions (being able to walk to a private place to be interviewed for 45 min), not having taken a sedative or any other medicine which influences consciousness, not having a history of a known psychological disorder, and being declared by their nurses to be physically and emotionally fit for an interview.

The inclusion criteria for the family caregivers (family members or relatives) consisted of being over 18 years old, being actively involved in their patient’s care, not having a known metabolic or psychological disorder, not having taken any medicine which affects the mind, not being physically or emotionally fatigued as a result of caring for their patient, and being prepared for a 45-min interview as confirmed by the interviewee. It was also necessary that the subjects be willing to participate and answer the questions to be included.

Data were collected through individual interviews, focus interviews, and field notes. Accordingly, 34 in-depth, semi-structured interviews were conducted with 34 participants on a face-to-face basis. Also, 2 focus group interviews were conducted with 2 groups consisting of 6 clinical nurses. All the interviews were carried out in the lecture halls of the hospitals or the nursing school with prior arrangements with the participants. The individual and focus group interviews lasted 45–70 and 60–90 min, respectively. The researchers also carried out observations in the hospital departments. Following each interview, the nurses’ interactions with the patients and family caregivers were observed and recorded. Moreover, during the interviews, the interviewees’ non-verbal communication was noted. Each field observation session lasted from 2 to 8 h and all the work shifts -morning, afternoon, and night- were included. In total, 6 observations, which lasted about 48 h, were carried out. The observations consisted of descriptions of the subjects, events, and nurses’ interactions with the patients and family caregivers.

Each interview (with the nurses, patients, and family caregivers) began with the general question “What is your understanding of the word “compassion?” followed by more specific questions. The specific questions for the nurses included: “What are your experiences of compassionate nursing care?”, “What are some examples of your caring behaviors which demonstrate compassion?”, “Can you talk about the role of compassion in your caring for patients during a work shift?”, “How does compassionate care influence your interactions with the patients and their family caregivers?”, and “When you speak about compassionate care, what are you reminded of?”

The specific questions for the patients included: “What are your experiences of compassionate nursing care during your stay in the hospital?”, “How do you feel when you receive nursing care combined with compassion?”, “How do you feel when you receive nursing care which is not accompanied by compassion?”, and “Based on your experiences, how do you define compassionate nursing care?”

The specific questions for the family caregivers included: “What are your experiences of compassionate nursing care during your patient’s stay in the hospital?”, “What is an example of compassionate nursing care given to your patient?”, “How do you feel when your patient receives nursing care combined with compassion?”, “How do you feel when your patient receives nursing care which is not accompanied by compassion?”, and “Based on your experiences, how do you define compassionate nursing care?” The researchers also asked some follow-up questions, e. g. “Can you explain further?”, “What do you mean by that?” and “Can you give an example?” in order to collect more information toward reaching the research objectives.

To ensure the rigor of the data collected in the qualitative phase, the researchers used the criteria suggested by Lincoln and Guba [24]. For credibility, the researchers used prolonged engagement with data, member checking, peer debriefing, triangulation of individuals (nurses, patients and family caregivers of different genders and age groups), and maximum variation sampling based on contrasting evidence. Dependability and confirmability were ensured through checking the accuracy of the transcripts and the extracted codes and categories by a panel of experts. To increase transferability, the researchers provided accurate and comprehensive descriptions of the concept in question, the participants’ characteristics and the manner of data analysis along with documented examples of the participants’ statements.

At the end of the qualitative phase, items for the questionnaire were developed based on the collected data. Next, using this template, the researchers created a pool of items based on the domains and sub-domains of the concept of compassionate nursing care (inductive approach). Also, the researchers conducted a review of literature and relevant questionnaires (deductive approach). The research team then merged the overlapping items, and the initial 80-item version of the questionnaire was considered for psychometric analysis. The initial draft was designed as a self-report questionnaire for measuring the nurses’ compassionate care.

### Phase 2. Quantitative study

The second phase of the study was an assessment of the psychometric properties of the instrument. Face and content validities were measured using qualitative and quantitative methods. Also, construct validity and reliability were measured.

### Face validity

For qualitative evaluation of face validity, the researchers assigned 12 nurses with different specialties, 3 nurse instructors, and 2 language experts to evaluate the items in terms of difficulty level, ambiguity, and syntax in face-to-face interviews. Their comments resulted in additions to the questionnaires’ content, but no item was deleted. Next, the quantitative method, based on the impact scores of the items, was used to evaluate the validity of the questionnaire. Accordingly, 10 nurses (2 from ICU, 2 from CCU, 1 from hemodialysis, 3 from internal, and 2 from surgical departments) who were working in the hospital were asked to comment on the importance of each of the items on a 5-point Likert scale (5 = very important, 4 = important, 3 = relatively important, 2 = not very important, 1 = not important at all). The impact score of each item was calculated and scores more than 1.5 were considered to be satisfactory [25]. The impact score coefficient was calculated using the formula below:

1 $$ \mathrm{The}\ \mathrm{impact}\ \mathrm{score}\ \mathrm{of}\ \mathrm{the}\ \mathrm{item}:\mathrm{Importance}\times \mathrm{Frequency}\left(\mathrm{percentage}\right)=\mathrm{Impact}\ \mathrm{score} $$

### Content validity

To test the content validity qualitatively, the researchers assigned 15 expert nurses (10 clinical nurses who were in practice in special care, internal and surgical departments and 5 doctor nurses who had extensive knowledge and experience in the field of instrument development and nurse education) to evaluate each item in terms of syntax, use of appropriate words, placement of the items and scoring and record their detailed comments in writing.

For quantitative evaluation of content validity, the content validity ratio (CVR) of each item was calculated to determine the necessity of that item. The content validity index (CVI) was used to examine the relevance of each item to the concept of compassionate care [26]. The Kappa coefficient for measuring agreement between the evaluators was calculated using the total content validity index (S-CVI) [27]. Content Validity Ratio (CVR) was rated on a 3-point Likert Scale (necessary, useful but not necessary, not necessary). According to Lawshe’s table, items with a score equal to or greater than 0.49 were retained [28]. The CVR of each item was calculated using the formula below:

2 $$ CVR=\frac{nE-\mathrm{N}/2}{N/2} $$

Content Validity Index (CVI) was calculated through Waltz and Bausell’s (2010) approach. Accordingly, 15 expert nurses were asked to evaluate the items in terms of relevance, simplicity and clarity on a 4-point Likert scale. The cutoff point for the CVI was set at 0.78 and higher [28]. Additionally, the Kappa statistics were calculated to determine the extent of agreement between the evaluators [27]. The mean of the content validity index (S-CVI) was used to calculate the total content validity index (S-CVI) [29]. The CVI of each item was calculated using the formula below:

3 $$ \mathrm{CVI}=\frac{\sum Number\ of\ answers\ 3\  or\ 4}{Total\ number\ of\ answers} $$

### Item analysis

Item analysis was performed to assess the Cronbach’s alpha coefficient for initial reliability and identify the items that affected the reliability [30, 31]. Before exploratory factor analysis, the items were analyzed with a sample of 40 clinical nurses who were selected via convenience sampling from special care (ICU, CCU, and emergency), internal and surgical departments. Most studies suggest a sample size of 30–50 subjects for item analysis [32]. The purpose of item analysis is to determine whether the items in a questionnaire are relevant to what it has been designed to measure or not [33]. Item analysis also aims to assess the correlation coefficients between the items: if an item does not have a correlation coefficient of at least 0.2–0.3 with at least another item, it should be omitted [34]. If an item has a correlation coefficient of more than 0.7 with another item, one of them should be omitted or they should be merged. Items with a total correlation coefficient of under 0.3 can also be omitted [35].

### Construct validity

In this study, the construct validity of the questionnaire was assessed via exploratory factor analysis. To determine the required sample size for factor analysis, 5–10 people per item have been recommended though larger sample sizes have also been suggested [36].

Boateng et al. (2018) suggest that the minimum sample size should be 300 to 450 subjects [37]. In the present study, 450 nurses were selected from different departments of university hospitals via convenience sampling. To collect data, the first researcher (BT) visited various departments (surgery, internal, ICU, CCU and emergency) of the hospitals on different days and at different shifts (morning, afternoon, and night). After obtaining permission from the supervisors and head nurses, the first researcher asked the nurses who met the inclusion criteria and were willing to participate in the study to complete the self-report compassionate care questionnaire. Response rate was 93.33%; of the 450 qualified nurses, 10 refused to participate due to work overload and fatigue, 6 were not willing to participate, and 14 failed to answer all the items on the questionnaire. Thus, in the end, 420 questionnaires were available for data analysis. The study population consisted of all the nurses who were in practice in the above-mentioned departments.

The inclusion criteria for the nurses were having at least a bachelor’s degree, willingness to participate in this research, those with manageable workload, and those in good physical and mental status. Those who failed to complete the questionnaires were fully excluded.

The exploratory factor analysis was performed using the Kaiser-Meyer-Olkin Index (KMO) and the Bartlett’s test of sphericity, main component analysis, scree plot and varimax Rotation with a sample size of 420 nurses. To determine the number of constructs, the researchers used initial eigenvalues and scree plot [38]. In the next step, exploratory factor analysis was performed using varimax rotation. The factor loading of each item in the factor matrix and the rotated matrix should be at least 0.4 [39].

In the second stage of evaluation of construct validity, to assess the final model of the factor construct of the questionnaire, the researchers conducted confirmatory factor analysis with a second sample consisting of 300 nurses. The analysis was completed using means and variance-adjusted weighted least square (WLSMV) in Mplus 6.1.

Confirmatory factor analysis is based on a theory and hypothesis test about the factor construct in question and is usually performed after determination of the correlation matrix or factor construct. In the present study, the most common goodness of fit models based on the accepted threshold were considered. The Chi-square goodness of fit, root mean square error of approximation (RMSEA), Tucker-Lewis Index (TLI), and comparative fit index (CFI) were calculated [40].

### Convergent validity

To evaluate convergent validity, we simultaneously distributed the present questionnaire and Caring Behaviors Inventory (CBI-42) developed by Wolf et al. (1998) among 100 nurses selected via convenience sampling from various departments (emergency, CCU, ICU, internal and surgery) of the university hospitals, and the correlation between their scores was measured.

### Reliability

The internal consistency and stability of the questionnaire were measured to assess its reliability. Internal consistency was assessed with a sample of 420 nurses. A Cronbach’s alpha of 0.7 to 0.8 indicated satisfactory internal consistency [31]. The test-retest method was used to assess the consistency of the questionnaire with 50 nurses over a two-week interval. The scores of the two tests were determined by calculation of the intra-class correlation coefficient (ICC) for each of the sub-domains and the whole questionnaire. Burns and Grow (2014) recommend that the stability of a questionnaire should be assessed over a period of 2 weeks in a month [41]. An ICC index rating of above 0.8 confirmed the stability of the instrument [42]. The ease of use of the questionnaire, as well as the ceiling and floor effects, were also studied. The latter were investigated using the same sample size used for evaluation of construct validity (420 subjects). Data analysis was performed using SPSS v.16.

## Results

In the qualitative stage, individual and focus group interviews were conducted to explain the concept of compassionate nursing care. Dimensions of the concept consisted of effective interaction, professionalism, and continuous comprehensive care. The definition of compassionate care as extracted from the interviews is as follows: compassionate care is professional care that takes place through clinical excellence, adherence to ethical values, and sensitivity to the needs. Effective interaction through emotional support, building trust and effective communication skills, along with continuous comprehensive care and attention to the patients’ existential dimensions, should occur at the same time [8].

At the end of the first phase, the nurses’ compassionate care questionnaire was developed according to the definition of the concept of compassionate nursing care and its constituent dimensions. The initial draft consisted of 98 items. During the review of available literature, 130 possible items were identified. The research team merged a number of overlapping items and the final number was reduced to 80.

### Psychometric properties (COSMIN criteria)

#### Face validity

Qualitative evaluation of face validity led to some modifications to and revisions of the items. For evaluation of quantitative face validity, the impact item scores were calculated and all the items, except for 5 items, were found to have a score of more than 1.5. Therefore, the number of items was further reduced from 80 to 75 items.

#### Qualitative content validity

In evaluation of qualitative content validity, the experts’ opinions led to the merging of overlapping items. Therefore, in the next stage, a questionnaire with 48 items was used to assess the quantitative content validity.

### Quantitative content validity [content validity ratio (CVR) and content validity index (CVI)]

After the CVR of the items had been calculated, 40 items had scores of more than 0.49 and were, therefore, retained. Given the cutoff point of 0.78 for the content validity index, no item was deleted as all of them were found to be above the minimum.

Also, the Kappa coefficient score for the 40 items was excellent. Mean score of the content validity index (SCVI/Ave) was 0.91, which is considered to be excellent.

### Item analysis

Before exploratory factor analysis, item analysis was performed with a sample size of 40 nurses. The reliability of the questionnaire was found to be 0.94 as measured through calculation of the Cronbach’s alpha coefficient. The results of item analysis to assess the correlation coefficients between the items and the total score led to omission of 2 items as decided by the research team. Then, the reliability of the questionnaire increased to 0.94. Thus, 38 items were left. As for the other items, each item correlated with at least one other item (0.2–0.3), and no item was deleted.

The mean age of the nurses was 27.58 ± 6.17 years and their mean work experience was 6.26 ± 4.77 years. The correlation coefficient of each item with the other items on the questionnaire was found to range between 0.21 and 0.7 and none of the items had a correlation coefficient of more than 0.7 with another item. Accordingly, none of the items was omitted or merged with another item in this stage. The correlation score of all the items with the total score of the questionnaire was found to be above 0.30.

### Hypothesis testing for construct validity

#### Sample size

To assess construct validity via exploratory analysis, the researchers had 420 nurses from various hospital departments complete the compassionate nursing care questionnaire. Table 1 shows the participants’ demographic characteristics. In the present study, the factor construct of the compassionate nursing care questionnaire was evaluated using exploratory factor analysis based on KMO sampling index and Bartlett’s test of sphericity, main component analysis, scree plot and Varimax rotation.

**Table 1:** Demographic characteristic of the Nurses in the construct validity section

Variable		Absolute distribution	Relative distribution(%)
Age	< 25	158	37.6
	26–35	206	49
	36–45	45	10.8
	46–55	11	2.6
Sex	Female	373	88.8
	Male	47	11.2
Education	Bachlor of degree	395	94
	Master of degree	25	6
Lenght of experience (years)	1–5	230	56.6
	6–10	118	26.5
	11–15	45	10.7
	16–20	17	3.8
	> 21	10	2.4
Ward	Surgical	146	34.5
	Internal	138	33.3
	I.C.U	85	20.2
	C.C.U	35	8.3
	Emergency	9	2.3
	Hemolysis	7	1.4

In evaluation of the construct validity of the questionnaire, the sampling adequacy index of Kaiser-Meyer-Olkin (KMO) was calculated and found to be 0.928. In addition, the result of Bartlett’s test of sphericity was significant (*P* < 0.001). The test showed that the chi-square of 4.785 with a degree of freedom of 528 was significant (P < 0.001). Thus, the results of Bartlett’s test confirmed those of the KMO test.

**Fig. 1:**
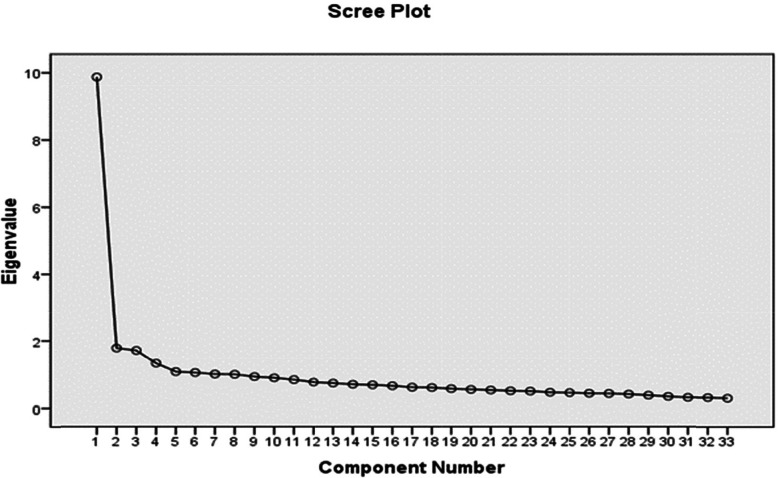
Scree plot to determine the number of factors in the questionnaire

To determine the number of the constructs in the questionnaire, the researchers employed the initial Eigenvalues and the scree plot. An initial analysis with a special value of more than one was performed, which, along with 8 factors, accounted for 57.278% of the observed variance. The scree plot showed that the major variance was due to the first factor, and it was flat for the other 4 factors (Fig. 1). Therefore, the number of factors was limited to 4 before factor analysis was performed. The results of factor analysis showed that 4 factors accounted for 48.05% of the variance.

In the next stage, exploratory factor analysis was performed using a varimax rotation. In the present study, a factor loading of 0.4 was considered the minimum acceptable degree of correlation between each item and the extracted factors. At this point, the items that had a high correlation with each other were put in the same category. The research team deleted five items that did not reach the minimum loading factor of 0.4 or had repetitive concepts.

The questionnaire ended up consisting of 28 items. To assess the goodness of fit of the final model of the factor construct of the 28-item scale, the researchers employed the goodness of fit chi-square confirmatory factor analysis test [*P* < 0.001, df = 347, *n* = 420, X^2^ = 723.185]. Next, the goodness of fit of the model was evaluated via other indexes. All the indexes of RMSEA = 0.051, CFI = 0.952 and TLI = 0.947 confirmed that the final model had satisfactory goodness of fit.

The final questionnaire had 28 items which addressed four factors of professional performance (9 items), continuous follow-up (6 items), patient-centered performance (7 items), and empathic communication (6 items) (Table 2). The manner of omission of the initial items (80 items) on the questionnaire through various stages of validity assessment is shown in additional file 1.

**Table 2:** Factor structure and factor loading of each item based on a varimax rotation

Domain	Item	Extracted factors			
		1	2	3	4
Professional performance	16. I am careful not to hurt my patients while taking care of them.	0.73			
	14. I respect my patients and their beliefs when I am giving nursing care.	0.67			
	12. When I am taking any clinical interventions, I respect the privacy of my patients.	0.59			
	15. I take care of my patients regardless of their economic, social, religious, and cultural status.	0.58			
	9. I take care of my patients according to medical principles.	0.57			
	30. I take the necessary measures to maintain my patients’ safety.	0.53			
	5. By giving professional care, I earn my patients’ confidence	0.51			
	13. I am careful to keep my patients’ information confidential.	0.50			
	23. My inner voice compels me to do my job well.	0.43			
Continuous follow-up	37. During my work shift, according to the conditions of my patients, I monitor them frequently by being present at their bedside.		0.66		
	36. I follow my patients’ care plans.		0.65		
	38. I report my patients’ complaints to the authorities.		0.58		
	35. I inform my patients and their family members about care and treatment		0.57		
	33. I encourage family members to emotionally support their patients.		0.55		
	32. I refer the patients in financial difficulties to a social worker or social support institution.		0.51		
Patient-centered performance	27. If my patients need it, I will devote some of my time, in addition to the routine visits, to their family members			0.64	
	10. Upon observing my patients’ conditions, I can identify their problems and take the necessary measures			0.63	
	11. I conduct nursing care planning (nursing diagnosis and prioritization of problems) on a regular basis.			0.56	
	18. My patients are entitled to accept or refuse treatment and care interventions.			0.53	
	29. I care about my patients’ spiritual needs.			0.49	
	26. I monitor the quality of my care daily.			0.47	
	17. I respect my patients’ independence.			0.42	
Empathic communication	3. To identify and solve my patients’ problems, I establish a sincere relationship with them within the cultural and religious framework.				0.69
	1. I provide care to my patients open-mindedly.				0.62
	2. When I am providing care, I empathize with my patients and their companions.				0.58
	4. By acting and speaking honestly, I try to win the confidence of my patients.				0.50
	7. I use my verbal communication skills (simple and clear speech and feedback) during care.				0.48
	8. Within the cultural and religious framework, I use non-verbal communication methods (e.g. eye contact and touch).				0.48

### Divergent construct validity

To examine the convergent validity of the instrument, the researchers simultaneously distributed the present questionnaire and the Caring Behaviors Inventory (CBI-42) developed by Wolf et al. (1998) among 100 nurses and measured the correlation between the respondents’ scores [43]. The correlation between the scores obtained with these two questionnaires was moderate (*p* < 0.001 and r = 0.68). The means of age and work experience of the nurses who participated in this stage were 28.3 ± 4.69 and 7.29 ± 5.77 years, respectively.

### Reliability (internal consistency and stability)

#### Internal consistency

For evaluation of internal consistency, the Cronbach’s alpha coefficients of the whole questionnaire and each of its domains (subscales) were calculated (Table 3).

**Table 3:** Cronbach’s alpha of subscales and the entire nurses’ compassionate care questionnaire

Factors	Subscale	Items	Cronbach’s alpha
1	Professional performance	9	0.83
2	Continuous follow-up	6	0.76
3	Patient-centered performance	7	0.73
4	Empathic communication	6	0.7
Entire	Questionnaire	28	0.89

#### Stability

To verify stability, the researchers employed the test-retest method. The scores of the two tests were determined via the calculation of the intra-class correlation coefficient (ICC) for each of the domains and the whole questionnaire (Table 4). The means of age and work experience of the nurses who participated in this stage were 25 ± 10.60 and 5.74 ± 4.54 years, respectively.

**Table 4:** Intra- cluster correlation between scores of subscales and total questionnaire of two tests

Factors	Subscales	ICC	Confidevce level of ICC (0.95)	*P*- value
1	Professional performance	0.91	0.84–84.95	*P* < 0.001
2	Continuous follow-up	0.85	0.74–0.91	*P* < 0.001
3	Patient-centered performance	0.86	0.72–0.92	*P* < 0.001
4	Empathic communication	0.88	0.78–0.94	*P* < 0.001
Entire	Questionnaire	0.94	0.89–0.96	*P* < 0.001

#### Measurement error

In the present study, absolute reliability was measured through calculation of standard error of measurement and standard error of mean (SEM). The results of standard error of measurement for the 4 subscales were 0.87, 1.01, 1.32 and 0.52, respectively.

#### Repeatability

In addition to stability, the researchers evaluated agreement. Agreement is considered to be positive when the smallest detectable change (SDC) or minimal detectable change (MDC) is greater than the minimal important change (MIC). As for the present questionnaire, the SDCs were greater than the MICs in all the domains. To assess agreement, the researchers first measured SEM. In addition, the split-half technique was used to assess the internal consistency. In the split-half method, the correlation coefficient between the first half and second half of a questionnaire are calculated. In the present study, the result was 0.82, indicating a satisfactory reliability of the questionnaire.

### Response rate

### Determination of the ease of use of the questionnaire

To assess the ease of use of the questionnaire, we calculated the average length of time needed for completing the questionnaire and the percentage of individuals who did not respond to each item. The average time needed to complete the questionnaire was found to be 4 min, with a range of 3–5 min. Also, for all items, the non-response rate should be 0–5%, as was the result with the present questionnaire.

### Determination of the ceiling and floor effects of the questionnaire

As to the ceiling and floor effects, more than 15% of the respondents obtained the highest or lowest possible scores. In general, the presence of a ceiling or floor effect indicates that the minimum or maximum severity of the phenomenon is not included in the questionnaire, which is a sign of poor content validity. The results of the present study regarding the ceiling and floor effects and construct validity (*n* = 420) showed that the minimum and maximum scores in none of the subscales and in the whole instrument reached 15%. Therefore, the questionnaire had no ceiling or floor effect.

The final version of the self-report compassionate nursing care questionnaire consists of 28 items. All the items are scored positively on a 5-point Likert scale: “always” (5 points), “often” (4 points), “sometimes” (3 points), “rarely” (2 points), and “never” (1 point) (Additional file 2). The score range of the questionnaire is between 28 and 140. The score range of each subscale is as follows: professional performance = 9–45, continuous follow-up = 6–30, patient-centered performance = 7–35, and empathetic communication = 6–30. Based on the differences between the subscales, the scores in the low third (28–65) are considered as poor, the scores in the middle third (66–103) as average, and those in the high third (104–140) as satisfactory.

The final version of the questionnaire consisted of 28 items on a 5-point Likert scale. Thus, the highest and lowest possible grades were 140 and 28 respectively. On a three-section range, evaluation of the scores was determined as follows:

$$ \mathrm{Determination}\ \mathrm{of}\ \mathrm{the}\ \mathrm{cut}\hbox{-} \mathrm{of}\mathrm{f}\ \mathrm{points}=\frac{\mathrm{maximum}\ \mathrm{score}-\mathrm{minimum}\ \mathrm{score}}{3} $$

Accordingly, the cut-off point for the present questionnaire was set at approximately 37; this amount was added to the minimum score (28) to determine the ranges. Thus, the scores in the lower third (28–65) were considered to be poor, those in the middle third (66–103) were regarded as average, and the scores in the top third (104–140) were considered to be satisfactory.

## Discussion

In the present study, the researchers developed an instrument for measuring compassionate nursing care and subsequently evaluated its psychometric properties. In the first stage, individual and focus group interviews were conducted to establish the meaning of the concept of compassionate nursing care as understood by nurses, nurse instructors, patients and family caregivers. The qualitative findings were classified into the three themes of effective interaction, professionalism and continuous comprehensive care. At the end of the qualitative phase, items were developed based on the practical definitions and a review of literature. Next, the psychometric properties of the developed questionnaire were assessed.

One of the important steps in developing a questionnaire is the process of item generation. In the present study, items were generated using a combination of inductive and deductive approaches. However, in some available instruments, the production of items has been solely through a review of literature and based on dictionary definitions (deductive approach) [20, 44, 45]. Also, due to the role of social and cultural factors in understanding the concept of compassionate care, there was a need to incorporate a deep understanding of the relevant experiences of nurses, patients and family caregivers.

Unlike the present questionnaire, the Schwartz Center compassionate care scale [17] and Fogarty’s Compassion Scale (1999) have been specifically designed for physicians [44]. Due to their different professional roles, physicians and nurses have different understandings of the needs of patients. Also, nursing care, which lasts longer, is of a different nature than medical care.

Some definitions of compassion which are based on dictionary classifications or literature reviews include references to empathy or sympathy [11], while compassionate care is conceptually broader than these concepts. Compassionate care emphasizes interventions for relieving suffering [2]. In some instruments, the word “compassion” itself is used instead of descriptive synonyms for compassion [17, 44, 45].

In the study of Lee and Simon (2017), the concept of compassion competence has been developed through analyzing a hybrid concept, and specific nursing behaviors are the basis of measuring the effects of compassion. In this study, only nurses in special care wards were interviewed [46], but in the present study, nurses, patients and family caregivers in various specialized units (CCU, ICU, hemodialysis, and emergency) and surgical and internal medicine were interviewed to determine the definition of the concept. Therefore, the present study contained richer information about the concept of compassionate care.

Compared to the present study, most previous studies have assessed face and content validity using a qualitative method only. In the study of Fogarty (1999) [44], evaluation of face and content validity was not carried out. The calculation of the item impact score, deletion of inappropriate items and determination of the importance of each item were executed, however [25]. Also, calculation of the content validity ratio in the present study helped identify the items that were necessary for measuring the concept [26]. Calculation of the content validity index helped identify the related concepts based on the opinions of experts [29]. The Kappa score of the questionnaire was excellent, indicating high inter-rater agreement over the relevance of the items.

In the present study, before the evaluation of construct validity, item analysis was performed. The results of the exploratory factor analysis indicated the adequacy of the sample size for construct validity assessment. Varimax rotation led to the classification of the 28 items into 4 domains: professional performance (9 items), continuous follow-up (6 items), patient-centered performance (7 items), and empathic communication (6 items). For evaluation of convergent validity, the researchers used the inventory of Wolf et al. (1998). The results showed a moderate correlation between Wolf’s scale and the present questionnaire.

The construct validity of the 18-item scale entitled the “Compassionate Competence Scale” developed by Lee and Simon (2016) was assessed via exploratory factor analysis on 660 nurses. It led to the placing of 17 individual items within one of the three factors of communication, sensitivity or insight. Evaluation of the convergent validity of the questionnaire showed a high correlation coefficient, but item analysis was not performed to identify the items affecting initial reliability [18].

In Grimani’s study (2017), the researcher’s manner of extracting factors and determining the factor structure in construct validity is not clear [47]. In the study of Burnell and Agan (2013), exploratory factor analysis with a sample of 250 hospitalized patients was conducted. The twenty-four items were divided into the four domains of meaningful relationship, patient expectations, care characteristics, and competent specialist. However, information on the adequacy of sampling is unavailable. Also, the methods of extracting the factors and determining the factor structure are not reported [45]. In some of the available tools, construct validity has not been assessed [20].

In the case of most of the existing tools, item analysis has not been performed to identify the items that affect reliability. In the present study, reliability was assessed through measurement of internal consistency and stability (test-retest method) in an interval of 2 weeks. As with the present study, the reliability of the nurses’ compassion competence scale was assessed through an examination of its internal consistency and test-retest [46]. In the present study, the half-split technique was also used to evaluate reliability. The linear correlation between the first half and the second half of the questionnaire indicated its satisfactory reliability.

In the case of most existing tools, stability has not been measured [19, 20, 44, 45]. The high stability of the present questionnaire shows that a respondent’s score on the test will remain constant over time, a feature which other questionnaires lack.

In the present study, the ceiling and floor effects of the developed questionnaire were studied on 420 nurses. One of the factors influencing the reliability of a tool is the ceiling and floor effects. If the effects do not exist, individuals with the highest and lowest scores cannot be evaluated and reliability decreases. No information on the ceiling and floor effects of the available tools has been reported.

In the present study, a broad spectrum of participants, including nurses, nurse educators, patients, and family caregivers with maximum variation, was studied followed by a comprehensive assessment of the psychometric properties of the questionnaire.

### Limitations

The development and evaluation of the psychometric properties of the present questionnaire were conducted in Iran. Therefore, it is recommended that further studies be carried out in other cultures, languages and contexts for cultural adaptation and more accurate evaluations of the reliability and validity of the questionnaire.

## Conclusion

A self-report questionnaire was designed in the present study to measure compassionate nursing care. The results of the study showed that the validity and reliability of the questionnaire were satisfactory. The questionnaire was also easy to use and could be completed quickly (approximately 4 min). Therefore, this is an appropriate questionnaire to measure the nurses’ compassionate care. Measuring compassion helps evaluate the performance of clinicians in terms of providing compassionate care and can enable nurse instructors and policymakers to adopt more effective strategies to promote compassionate care, an important aspect of holistic care.

## Supplementary Information

**Additional file 1.** The development and elimination of items in each stage of validity assessment.

**Additional file 2.** Compassionate Care Questionnaire for Nurses.

## Data Availability

The interview data will not be shared since the participants are guaranteed full anonymity.

## Abbreviations

COSMIN: Consensus-based Standards for the selection of health Measurement Instrument
CCU: Coronary Care Unit
ICU: Intensive Care Unit
CVR: Content Validity Ratio
CVI: Content Validity Index
S-CVI: Scale-level content validity index
S-CVI /Ave: Scale-level content validity index/ Average
KMO: Kaiser-Meyer-Olkin Index
WLSMV: Mean and variance-adjusted weighted least square
RMSEA: Root mean square error of approximation
TLI: Tucker-Lewis Index
CFI: Comparative Fit Index
CBI-42: Caring Behaviors Inventory- 42 items
ICC: Intra-class correlation coefficient
SEM: Standard Error of Mean
SDC: Smallest Detectable Change
MDC: Minimal detectable change
MIC: Minimal Important Change
