# Supplementary material for: Development and assessment of the psychometric properties of a compassionate care questionnaire for nurses
Source: BMC Nurs. 2021 Oct 7;20:190. doi: 10.1186/s12912-021-00691-3 (PMC8495991; doi:10.1186/s12912-021-00691-3)
Supplement: Supplementary file 1 — Additional file 1. The development and elimination of items in each stage of validity assessment. [file 12912_2021_691_MOESM1_ESM.docx]

**Additional file 1. The development and elimination of items in each stage of validity assessment**

| **Exploratory Factor Analysis** | **Item Analysis** | **CVI** | **CVR** | **Qualitative content analysis** | **Impact**  **Factor** | **Items** |  |
| --- | --- | --- | --- | --- | --- | --- | --- |
| 0.62 | >0.3 | 1 | 0.73 | - | 5 | I provide care to my patients open-mindedly. | 1 |
| - | - | - | - | - | 1.04  (omitted) | At the time of change of shift, I greet my patients warmly. | 2 |
| - | - | - | 0.33  (omitted) | - | 3.78 | When I feel that my patients are suffering, I offer a solution. | 3 |
| - | - | - | - | merged with item 6 | 4.14 | I pay attention when my patients talk to me about their troubles. | 4 |
| <0.4  (omitted) | >0.3 | 1 | 0.6 | edited | 3.76 | When I realize my patients’ problems, I try to solve them. | 5 |
| 0.58 | >0.3 | 1 | 0.68 | - | 4.7 | When I am providing care, I empathize with my patients and their companions | 6 |
| 0.69 | >0.3 | 1 | 0.73 | - | 3.28 | To identify and solve my patients’ problems, I establish a sincere relationship with them within the cultural and religious framework. | 7 |
| - | - | - | 0.43  (omitted) | edited | 3.08 | I share strategies I have learned from my personal experiences with my patients. | 8 |
| - | - | - | - | merged with item 7 | 3.28 | To identify and solve my patients’ problems, I establish a friendly relationship with them. | 9 |
| - | - | - | 0.46  (omitted) | - | 3.2 | To make my patients feel good, I use humor within the boundaries of my profession. | 10 |
| - | - | - | 0.4  (omitted) | - | 3.25 | I answer the questions of my patients and their companions kindly. | 11 |
| 0.50 | >0.3 | 1 | 1 | edited | 4.05 | By acting and speaking honestly, I try to win the confidence of my patients. | 12 |
| 0.51 | >0.3 | 1 | 0.6 | - | 4.7 | By giving professional care, I earn my patients’ confidence | 13 |
| - | - | - | 0.35  (omitted) | - | 4.32 | I let my patients participate in their care and treatment process. | 14 |
| - | - | - | - | - | 1.05  (omitted) | To confirm my patients’ feelings, I pat them on the shoulder. | 15 |
| 0.48 | >0.3 | 1 | 0.6 | edited | 3.44 | I use my verbal communication skills (simple and clear speech and feedback) during care. | 16 |
| <0.4  (omitted) | >0.3 | 1 | 0.86 | - | 5 | I listen to my patients carefully. | 17 |
| <0.4  (omitted) | >0.3 | - |  | merged with item 16 | 4.8 | I speak to my patients in simple words, in an honest manner, and in a proper tone. | 18 |
| 0.48 | >0.3 | 1 | 0.73 | - | 4.7 | Within the cultural and religious framework, I use non-verbal communication methods (e.g. eye contact and touch). | 19 |
| - | - | - | - | merged with item 19 | 4.6 | Through non-verbal communication, I show my kindness to my patients. | 20 |
| <0.4  (omitted) | >0.3 | 1 | 0.86 | - | 4.14 | I try to adjust my care to my patients’ needs. | 21 |
| 0.57 | >0.3 | 1 | 0.86 | - | 4.9 | I take care of my patients according to medical principles. | 22 |
| - | - | - | - | - | 1.02  (omitted) | I inform my patients about the various aspects of their care and treatment. | 23 |
| 0.63 | >0.3 | 1 | 0.73 | - | 3.52 | Upon observing my patients’ conditions, I can identify their problems and take the necessary measures. | 24 |
| 0.56 | >0.3 | 1 | 0.6 | - | 5 | I conduct nursing care planning (nursing diagnosis and prioritization of problems) on a regular basis. | 25 |
| - | - | - | - | merged with item 21 | 4.14 | I adjust my care to my patients’ characteristics. | 26 |
| - | - | - | - | merged with item 74 | 4.8 | To maintain my patients' peace, I control the sound of alarms and  phone ringtones. | 27 |
| - | - | - | 0.41  (omitted) | - | 4.9 | I treat my patients’ information as professional secrets. | 28 |
| - | - | - | - | merged with item 21 | 4 | When providing care, I consider all the needs of my patients. | 29 |
| 0.50 | >0.3 | - | - | - | 4.9 | I am careful to keep my patients’ information confidential. | 30 |
| 0.67 | >0.3 | 1 | 0.86 | - | 4.8 | I respect my patients and their beliefs when I am giving nursing care. | 31 |
| - | - | - | 0.38  (omitted) | - | 1.4 | I talk to my patients' families to get them to treat the patients lovingly. | 32 |
| 0.58 | >0.3 | 1 | 0.73 | - | 4.9 | I take care of my patients regardless of their economic, social, religious, and cultural status. | 33 |
| - | - | - | 0.2  (omitted) | - | 4.23 | I try to give equitable care to all my patients. | 34 |
| 0.73 | >0.3 | 1 | 1 | - | 4.9 | I am careful not to hurt my patients while taking care of them. | 35 |
| 0.59 | >0.3 | 1 | 1 | - | 4.8 | When I am taking any clinical interventions, I respect the privacy of my patients. | 36 |
| - | - | - | 0.33  (omitted) | - | 4 | I am careful to treat my patients’ medical information as confidential. | 37 |
| 0.42 | >0.3 | 1 | 0.73 | - | 3.96 | I respect my patients’ independence | 38 |
| 0.53 | >0.3 | 1 | 0.6 | - | 3.96 | My patients are entitled to accept or refuse treatment and care interventions. | 39 |
| - | - | - | - | merged with item 33 | 4 | I do not ridicule my patients’ beliefs and values. | 40 |
| <0.4  (omitted) | >0.3 | 1 | 0.86 | - | 5.9 | At the beginning of my shifts, I introduce myself and explain my duties to my patients. | 41 |
| <0.4  (omitted) | >0.3 | 1 | 0.73 | - | 4.05 | In my care plans, I allocate time to answering my patients’ questions. | 42 |
| - | - | - | - | merged with item 25 | 4.23 | I schedule my care plan regularly***.*** | 43 |
| <0.4  (omitted) | >0.3 | 1 | 0.6 | - | 2.73 | In all situations at work, my first priority is providing optimal care to my patients | 44 |
| <0.4  (omitted) | >0.3 | 1 | 0.73 | - | 2.73 | I inform my patients about their illness, possible treatments and  their medicine. | 45 |
| <0.4  (omitted) | >0.3 | 1 | 0.6 | - | 5 | I believe that if I do my job well, I will be rewarded by God. | 46 |
| <0.4  (omitted) | >0.3 | 1 | 0.86 | - | 5.9 | I concern myself with the patients’ problems | 47 |
| 0.43 | >0.3 | 1 | 0.6 | - | 2.59 | My inner voice compels me to do my job well | 48 |
| - | - | - | 0.46  (omitted) | - | 2.66 | I inform my patients about their illness and treatment plan within my professional boundaries. | 49 |
| - | - | - | 0.3  (omitted) | - | 4.23 | In difficult conditions in the ward, my top priority is providing optimal care to my patients. | 50 |
| - | - | - | 0.31  (omitted) | edited | 2.73 | When I am treated harshly by my patients or their companions, I remain patient. | 51 |
| <0.4  (omitted) | >0.3 | 0.93 | 0.6 | edited | 3.52 | When caring for my patients, I consider their physical, psychological, spiritual, social and cultural needs. | 52 |
| - | - | - | 0.33  (omitted) | merged with item 52 | 4.14 | When giving care, I consider my patients’ cultural values and beliefs***.*** | 53 |
| - | - | - | 0.4  (omitted) | - | 2.08 | I introduce my patients to sources and organizations of support as necessary. | 54 |
| - | - | - | 0.3  (omitted) | - | 3.36 | When performing any nursing intervention, I make sure that my patients’ bodies are properly covered. | 55 |
| 0.64 | >0.3 | 1 | 0.6 | - | 4.8 | If my patients need it, I will devote some of my time, in addition to the routine visits, to their family members | 56 |
| - | 0.2  (omitted) | 0.8 | 0.73 | - | 3.38 | I encourage my patients to express their feelings about their illness and treatment. | 57 |
| <0.4  (omitted) | >0.3 | 1 | 0.73 | - | 2.03 | I believe that the emotional support of my patients’ families helps their treatment. | 58 |
| - | - | - | - | merged with item 56 | 2.66 | The companion is allowed to be with the patient. | 59 |
| 0.49 | >0.3 | 1 | 0.86 | - | 5.8 | I care about my patients’ spiritual needs. | 60 |
| - | - | - | 0.46  (omitted) | - | 5.8 | I help my patients perform their religious activities as they choose. | 61 |
| 0.53 | >0.3 | 1 | 0.86 | - | 5.8 | I take the necessary measures to maintain my patients’ safety | 62 |
| - | - | - | 0.2  (omitted) | - | 4.01 | I wash my hands before and after touching my patients. | 63 |
| - | - | - | 0.3  (omitted) | - | 4.14 | I talk to my patients' families to get them to treat the patients lovingly. | 64 |
| 0.47 | >0.3 | 1 | 0.6 | - | 2.08 | I monitor the quality of my care daily. | 65 |
| - | 0.21  (omitted) | 0.78 | 0.86 | - | 4.6 | I try my best to satisfy the desires of my terminally ill patients. | 66 |
| - | - | - | 0.38  (omitted) | - | 3.01 | I try to provide my terminally ill patients with peace. | 67 |
| 0.51 | - | 1 | 0.73 | - | 4.7 | I refer the patients in financial difficulties to a social worker or social support institution. | 68 |
| - | - | - | 0.46  (omitted) | - | 4.9 | I actively try to keep my patients’ information confidential. | 69 |
| - | - | - | - | - | 1.05  (omitted) | By providing emotional care, I make stressful conditions more tolerable. | 70 |
| 0.55 | >0.3 | 1 | 0.73 | - | 2.36 | I encourage family members to emotionally support their patients | 71 |
| - | - | - | 0.41  (omitted) | - | 2.03 | To help my patients heal, I consider the emotional support of their families. | 72 |
| - | - | - | - | - | 1.05  (omitted) | To increase my patients’ comfort, I make sure that their environment is clean | 73 |
| - | - | - | 0.3  (omitted) | - | 5 | To maintain my patients’ peace, I make sure that their environment is properly-ventilated, clean and quiet. | 74 |
| - | - | - | 0.34  (omitted) | - | 4.9 | If my patients do not receive a treatment or proper care, I look into the reasons. | 75 |
| 0.57 | >0.3 | 1 | 0.86 | - | 4.9 | I inform my patients and their family members about care and treatment. | 76 |
| 0.65 | >0.3 | 1 | 0.6 | - | 4.9 | I follow my patients’ care plans. | 77 |
| - | - | - | - | merged with item 36 | 3.01 | My examination should be done in a private environment. | 78 |
| 0.66 | >0.3 | 1 | 0.6 | - | 4.9 | During my work shift, according to the conditions of my patients, I monitor them frequently by being present at their bedside | 79 |
| 0.58 | >0.3 | 1 | 0.6 | - | 4.14 | I report my patients’ complaints to the authorities. | 80 |
