# Supplementary material for: Development and assessment of the psychometric properties of a compassionate care questionnaire for nurses
Source: BMC Nurs. 2021 Oct 7;20:190. doi: 10.1186/s12912-021-00691-3 (PMC8495991; doi:10.1186/s12912-021-00691-3)
Supplement: Supplementary file 2 — Additional file 2. Compassionate Care Questionnaire for Nurses. [file 12912_2021_691_MOESM2_ESM.docx]

**Additional file 2: Compassionate Care Questionnaire for Nurses**

| **Domain** | **Item** |  |  |  |  |  |
| --- | --- | --- | --- | --- | --- | --- |
|  |  | **Always** | **Often** | **Sometimes** | **Rarely** | **Never** |
| Professional performance | 1. I try not to hurt the patient while taking care of him/her. |  |  |  |  |  |
|  | 2. I respect the patient and his/her beliefs in nursing care. |  |  |  |  |  |
|  | 3. When I take any clinical intervention, I take into consideration the privacy of the patient |  |  |  |  |  |
|  | 4. I take care of my patient regardless of the economic, social, religious, and cultural conditions |  |  |  |  |  |
|  | 5. Based on scientific principles, I take care of my patient. |  |  |  |  |  |
|  | 6. I take necessary measures to maintain patient safety. |  |  |  |  |  |
|  | 7. By taking expert care, I attract my patient’s confidence |  |  |  |  |  |
|  | 8. I'm trying to keep my patient information safe. |  |  |  |  |  |
|  | 9. My inner strength obliges me to do my care well. |  |  |  |  |  |
| Continuous follow up | 10. During the work shift, according to the conditions of the patient, I monitor him/her with a more frequency at the bedside. |  |  |  |  |  |
|  | 11. I follow my patient care affairs. |  |  |  |  |  |
|  | 12. I report my patient complaints to related authorities |  |  |  |  |  |
|  | 13. I train the patient and his family members about care and treatment |  |  |  |  |  |
|  | 14. I encourage family members to emotionally support their patients. |  |  |  |  |  |
|  | 15. In case of a financial problem, I refer the patient to a social worker or related social support institutions. |  |  |  |  |  |
| Patient-centered performance | 16. If I need a patient, I will consider a time in addition to the routine visit times for family members |  |  |  |  |  |
|  | 17. Upon observing the patient's condition, I can diagnose her/his problems and take necessary measures |  |  |  |  |  |
|  | 18. I conduct nursing care planning (nursing diagnosis and prioritization of problems) on a regular basis. |  |  |  |  |  |
|  | 19. My patient is entitled to accept or refuse treatment and care interventions |  |  |  |  |  |
|  | 20. I care about my patients’ spiritual needs. |  |  |  |  |  |
|  | 21. I monitor the quality of my daily care. |  |  |  |  |  |
|  | 22. I try to care for the patient's independence |  |  |  |  |  |
| Empathic communication | 23. To identify and solve my patients’ problems, I establish a sincere relationship with him/her in the cultural and religious framework |  |  |  |  |  |
|  | 24. With open-mindness, I provide care to the patient |  |  |  |  |  |
|  | 25. When I am providing care, I empathize with my patients and their companions. |  |  |  |  |  |
|  | 26. With honesty in my behavior and speech, I try to win the confidence of my patient |  |  |  |  |  |
|  | 27. use verbal communication skills (simple and clear speaking and feedback) during care |  |  |  |  |  |
|  | 28. In the cultural and religious context, I use non-verbal communication methods (eye contact, touching, and face-to-face). |  |  |  |  |  |
